# Ecologic and Sociodemographic Risk Determinants for Dengue Transmission in Urban Areas in Thailand

**DOI:** 10.1155/2012/907494

**Published:** 2012-09-26

**Authors:** Surachart Koyadun, Piyarat Butraporn, Pattamaporn Kittayapong

**Affiliations:** ^1^Center of Excellence for Vectors and Vector-Borne Diseases, Faculty of Science, Mahidol University, Nakhon Pathom 73170, Thailand; ^2^Department of Biology, Faculty of Science, Mahidol University, Bangkok 10400, Thailand

## Abstract

This study analyzed the association between household-level ecologic and individual-level sociodemographic determinants and dengue transmission in urban areas of Chachoengsao province, Thailand. The ecologic and sociodemographic variables were examined by univariate analysis and multivariate logistic regression. In the ecologic model, dengue risk was related to households situated in the ecotope of residential mixed with commercial and densely populated urban residential areas (RCDENPURA) (aOR = 2.23, *P* = 0.009), high historical dengue risk area (aOR = 2.06, *P* < 0.001), and presence of household window screens (aOR = 1.62, *P* = 0.023). In the sociodemographic model, the dengue risk was related to householders aged >45 years (aOR = 3.24, *P* = 0.003), secondary and higher educational degrees (aOR = 2.33, *P* = 0.013), household members >4 persons (aOR = 2.01, *P* = 0.02), and community effort in environmental management by clean-up campaign (aOR = 1.91, *P* = 0.035). It is possible that the preventive measures were positively correlated with dengue risk because these activities were generally carried out in particular households or communities following dengue experiences or dengue outbreaks. Interestingly, the ecotope of RCDENPURA and high historical dengue risk area appeared to be very good predictors of dengue incidences.

## 1. Introduction

Dengue virus, an *Aedes* mosquito-borne viral pathogen belonging to the family Flaviviridae, is the cause of dengue fever (DF) and dengue hemorrhagic fever (DHF). The emergence and reemergence of DF/DHF have become a significant public health burden in the tropics and subtropics [1–5]. Due to the lack of an effective tetravalent dengue vaccine that can secure lifelong immunization, *Aedes* mosquito control measures have primarily been employed to prevent disease outbreak and interrupt transmission during the outbreak [6]. Regarded as a reemerging infectious disease [1, 4, 5], intermittent epidemics of DF and DHF have occurred in vulnerable populations. Such outbreaks reflect the failure of current prevention and control efforts, despite the fact that some successful cases of vector control in the Americas, Cuba, and Singapore had shortened outbreak periods and stopped the diseases from spreading [7–9]. In some cases, an application of appropriate vector control measures along with community participation have proven more effective and sustainable than antimosquito approaches alone [10–12]. 

Several reports have shown a coherent argument that transmission dynamics of dengue viruses result from very complex epidemiology and ecology of the disease. Such dengue transmission dynamics are the interaction among humans, dengue viruses, vectors, and ecosystems, of which biotic and abiotic determinants have both direct and indirect influences on dengue transmission [13–16]. Obviously in some cases, a useful set of environmental and sociodemographic factors, which constituted age-dependent classes, numbers and densities of urban populations, economic classes, and inhabitations, are central components of analysis of temporal and spatial relationships of dengue incidences [17–20]. Also, a vector-based dengue model [21] can predict transmission dynamics, based primarily on the infestation and/or reinfestation of domestic and peridomestic *Aedes* mosquito vectors in human inhabitations. Nonetheless, in different complex epidemiological settings, various factors that can influence dengue transmission dynamics remain to be established. This is because of more diverse sociocultural contexts and changes in sociopolitic, socioeconomic, demographic, technologic, and environmental conditions as well as ineffective management of household-level information and improper implementation of those control strategies.

Investigation into such sociodemographic, environmental perspectives can provide foresight into the appropriateness of dengue control efforts, give answers to unexpected vector control responses, and contribute to effective management solutions in an ever-changing environment. Of note, a plausible paradigm of interdisciplinary approach that integrates the ecologic and sociodemographic dimensions of dengue [22–27] has permitted an analysis of dengue transmission risk to determine what pivotal drivers significantly contribute to dengue transmission in an urban environment. In this regard, we applied two sets of household-level ecologic and individual-level sociodemographic factors to determine whether two fitted models of dengue-related determinants predicted the dengue transmission risk in urban areas of Chachoengsao province, Thailand, known for epidemics of DF/DHF [12]. Ultimately, the findings of this study would benefit better management of effective dengue prevention and control, especially in resource-limited developing countries. 

## 2. Materials and Methods

### 2.1. Study Area and Household Selection

**Figure 1:**
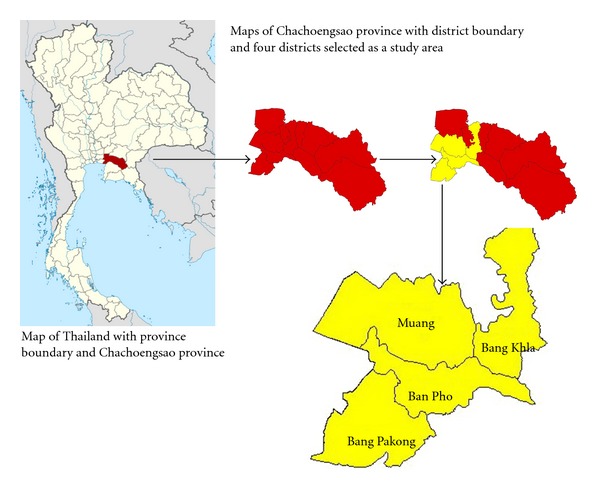
Maps of study area: 4 districts including Muang, Bang Pakong, Ban Pho, and Bang Khla. Map of Thailand obtained from http://www.wikimedia.org/.

Chachoengsao province was a representative of geographically defined areas (Figure 1), including small-and medium-sized municipalities as well as four main types of landscapes: mountains, rivers, flatlands, and wetlands. Both urban and semiurban communities are situated in low-lying, generally flat areas surrounded by rice fields and orchards. In the area, the climate is characterized by a long rainy season (June–October), a winter dry season (November–January), and a hot dry season (February–May). Given the complexity of its demographic and socioeconomic dispersions, the province is administratively divided into 11 districts covering a total area of 5,370 km^2^ with basic infrastructures of connecting roads, electricity, piped water supply system, communication system, and health service system. 

**Figure 2:**
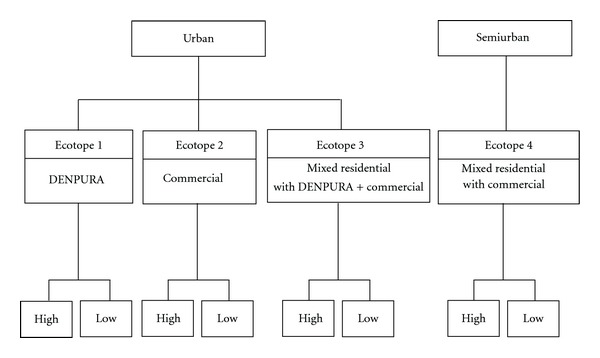
Diagram showing selection criteria for the 12 blocks: (1) degree of urbanization and (2) intensity of dengue transmission. Abbreviation: DENPURA—densely populated urban residential area.

 Two-stage random sampling was applied for selecting targeted households. The 120 blocks initially assigned to four different districts were used to select 12 blocks (approximately 100 houses each), based on the degree of urbanization and the intensity of dengue transmission (Figure 2). Six urban blocks were selected from the municipality of Muang district and the capital of Chachoengsao province, while the other six semiurban blocks were chosen from three subdistrict municipalities: Bang Pakong, Ban Pho, and Bang Khla. According to this household selection, a sample size was calculated based on 95% confidence to detect the prevalence of IgG-IgM-positive school children at 19.2% in Chachoengsao province [12] with a 3% of accepted error. The statistically required sample size of 994 was increasing by 20% to allow for missing data, resulting in 1,200 households, which were derived from the 12 selected blocks. All were subsequently used for household surveys during August–October 2007 to collect household-level and individual-level information with the assistance of interdisciplinary teams (i.e., each team included well-trained professional nurses and/or public health officers), as described below.

The establishment of interdisciplinary approaches and teams was supported by a WHO/TDR/IDRC-funded multicountry study. In this study, the instruments provisionally guided by multicountry supporting teams were initially developed from a series of community of practice workshops delivered through a multicountry network in Asia. The tools were partly modified with the addition of a useful set of variables, which corresponded to sociocultural contexts of Thailand. As technically validated by the authors, all tools included the structured questionnaires on households and individuals along with the environmental observation checklist. 

### 2.2. Ecologic Factors of Households

The structured questionnaires on households along with the environmental observation checklist were used to gather a set of ecological data of the entire 1,200 households. The data included ecotope, dengue risk area, number of house floors, floor of principal living (i.e., homeowners or any member often used a space of everyday family living as principal living area of the first floor or the upper floors), construction material of the house, number of house windows, having window screens, having a yard/open space, having bushes in a yard/open space, main purpose of house, and household attachment (i.e., the attached houses had at least one shared wall, whereas the detached houses had standalone property with no sharing wall). Face-to-face interviews were carried out using the representative respondents who were family members, 18 years of age or older. 

**Figure 3:**
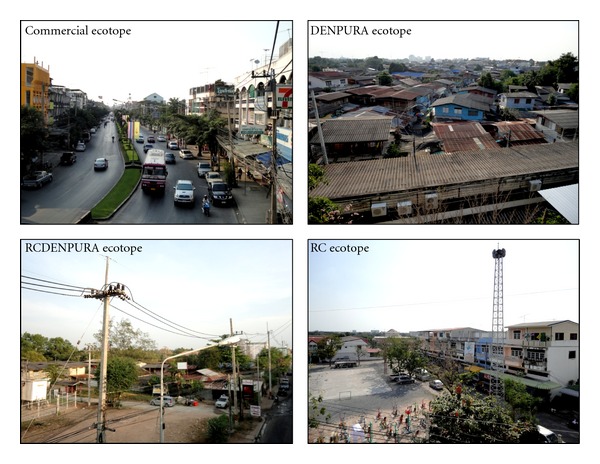
The four different characterized ecotopes.

In this study, we applied an ecotope concept, which is originally defined as the smallest ecologically-distinct features in a landscape mapping and classification system. As mentioned earlier, those selected 12 blocks were assigned to have 4 different ecotopes (Figure 3), which spanned both urban and semiurban blocks of the study area. The difference is based quantitatively and qualitatively on the distribution of houses, the socioeconomic status (SES) of local inhabitants, infrastructural services, land use, and land type. The commercial ecotope (C) denoted a majority of attached houses with a 0.07-meter mean distance of nearest houses, a high SES of occupants, and good basic infrastructure, along with a great deal of commercial and business buildings. The ecotope of a densely populated urban residential area (DENPURA) is characterized by most attached houses with a 0.63-meter mean distance of nearest houses, low SES of the population, and relatively low degree of community development, including social and economic opportunities and access to health care resources as well as infrastructural water supply and waste management and a lack of vegetation and/or forested areas. The ecotope of residential (R) mixed with C and DENPURA (RCDENPURA) exhibits a main residential zone prominently scattered with C and DENPURA and has a 3.37-meter mean distance of nearest houses, while the residential mixed only with commercial (RC)—signifying an overlap of urban and rural areas—is an area mainly for dwelling but which is also filled in partly with commercial settlements and has a 4.58-meter mean distance of nearest houses. Finally, each ecotope investigated in this study had a different number of study households—C (*n* = 200), DENPURA (*n* = 200), RCDENPURA (*n* = 200), and RC (*n* = 600). 

As for the risk area, the classification of historical dengue risk areas (high and low degrees of dengue transmission) was drawn from which its epidemic pattern that normally occurs on a 2-3 year-cycle transmission [28]. Therefore, based on the past situation of national surveillance of dengue cases, the definition of dengue transmission risk areas relies upon a 3- to 5-year median of dengue cases. In this study, the data of the confirmed dengue cases retrieved from the national epidemiological surveillance system were obtained from the Chachoengsao General Hospital and Chachoengsao Provincial Public Health Office [29]. Therefore, based on the ecotope assignment (Figure 2), the study households (*N* = 1,200 households) were categorized into 2 historical dengue risk areas: 6 high-transmission blocks (*n* = 600 households) that had the highest 5-year medians of dengue cases and 6 low-transmission blocks (*n* = 600 households) that had the lowest 5-year medians of dengue cases.

### 2.3. Sociodemographic Factors of Respondents

The structured questionnaires on individuals, which had a significant reliability of knowledge, attitude, and practice (Cronbach's alpha coefficient = 0.7), were used to gather a set of sociodemographic data using those representative respondents of the entire 1,200 households as previously mentioned. The data included age, education level (highest school degree), occupation, movement during the last 3 months, residence time in house, residence time in neighborhood, number of household members, average family income, knowledge about dengue and its vectors, attitude about dengue vector control in terms of gender, family, and government roles, household water storage (i.e., by using any water-storing containers), household practices to reduce the nuisance of mosquitoes, household practices to prevent *Aedes *breeding places, last time visited by health personnel, receiving dengue control support/materials, and community efforts in environmental management by clean-up campaign.

 Regarding household practices to reduce the nuisance of mosquitoes, questions were asked about household activities which could be categorized into 3 groups: (1) chemical control (e.g., indoor spraying, putting chemicals in water containers and personal protection with repellents), (2) physical control (e.g., removing rubbish, covering water containers and killing mosquitoes), and (3) biological control (e.g., putting fish in water containers). The chemical and physical control activities were ranked based on frequent actions: low (never or either one of three actions) and high (two or more actions), whereas the biological control activity was ranked based on the action “No or did not apply” and “Yes or applied.” 

The questions of household practices to prevent *Aedes *breeding places could be categorized into 3 groups: (1) chemical control (e.g., putting chemicals in water containers), (2) physical control (e.g., removing rubbish, covering the water containers, changing water once a week, eliminating stagnant water, brushing/cleaning inner surface of water containers, and removing larvae), and (3) biological control (e.g., putting fish in water containers). The chemical and biological control activities were ranked as either “Yes” or “No.” The physical control activities were ranked based on the frequency of actions: low (never, one, or two actions) and high (three and more actions). 

All the respondents provided informed consent after they were completely informed about the study's purpose, as well as the advantage and disadvantage of participation. The ethical clearance for this study was approved by the Institutional Review Board of Mahidol University. 

### 2.4. Data Analysis

Dengue-related determinants as the outcomes of dengue transmission risk in the study area included the households that had past history of dengue cases and the respondents or householders that had history of dengue infections in their lifetime before the study. Generally speaking, the principal outcome of this study was to identify whether those household-level ecologic and individual-level socioeconomic variables were correlated to these dengue-related determinants. Therefore, in two separate ecological and socioeconomical models, a univariate analysis of the individual explanatory variables was used to analyze the association of the dengue-related determinants using the chi-square test (*P* < 0.05 or *P* < 0.1). Then, they were entered into the multivariate logistic models and odds ratios (aORs) adjusted for all of these variables and 95% confidence intervals (CI) were calculated. In fitted models, a pseudo *R* ^2^ value was considered when the regression model was sufficiently adequate. The likelihood-ratio (LR) test (*P* < 0.05) was used to test the significance of all the related predictors while the Wald's test (*P* < 0.05) was used to test the statistical significance of each coefficient (*b*) in the model to determine contributing predictors. The SPSS statistical program, version 17.0 (SPSS, Inc., Chicago, IL, USA), was used throughout this study. 

## 3. Results and Discussion

### 3.1. Ecologic Risk Factors for Dengue Transmission

We attempted to directly relate household-level ecologic determinants with dengue transmission risk.  Table 1 reveals the results of the univariate analysis of ecologic risk factors for dengue transmission considering the household-level characteristics. Using *χ* ^2^ test, 4 out of 11 ecologic variables were found to have a significant association (*P* < 0.05) with frequency of houses having previous history of dengue cases. Four significant variables included ecotope, historical dengue risk area, number of house windows, and presence of screens for house windows. The three variables of ecotope, historical dengue risk area, and presence of screens for house windows were selected for the final multivariate regression while the number of house windows was not entered in the model because it appeared to be of marginal significance. Results of the multivariate analyses are shown in Table 2. In multivariate analysis, these selected determinants were adjusted for their confounding factors by the random effect model. All determinants (the ecotope, historical dengue risk area, and presence of screens for house windows) seemed to have a direct impact on dengue transmission (the LR test, *P* < 0.05). For the ecotope, three categorical variables (DENPURA, RCDENPURA, and RC) were used to determine their effect on dengue transmission using the commercial ecotope as a reference. Only the ecotope of RCDENPURA remains as a significant predictor for dengue transmission (aOR = 2.23, *P* = 0.009). In terms of dengue risk area, a setting with a high degree of historical dengue transmission indicated OR (aOR = 2.06, *P* < 0.001) significantly higher than an area with a low degree of historical dengue transmission. The last significant variable of presence of screens for house windows showed that houses with window screens had a greater risk (aOR = 1.62, *P* = 0.023) than those with no window screens. 

Dengue is primarily a mosquito-borne disease found in urban and semiurban settings. The morbidity and mortality attributed to this disease may vary significantly from one place to another on account of different local parameters. In fact, many relevant studies have demonstrated that it is the set of microsocioeconomic, infrastructural, and environmental parameters embedded in communities which appear to be responsible for increased epidemic transmission of dengue virus in particular ecosystem localities [22, 30–34]. Ecologic factors investigated in this study are key determinants for dengue transmission risk. In addition, a novel ecosystem concept was created to exhaustively scrutinize the associations between ecotope and the transmission of dengue in the study areas. Of all 4 ecotopes, only the ecotope of RCDENPURA (i.e., where the ecotope is in fact a combination of residential, commercial, and densely populated urban residential areas) exhibited the strongest association with dengue transmission suggesting that the complexity of urban ecosystem may give rise to dengue emergence [13]. To our knowledge, this is the first time that the ecotope, which greatly poses the most risk for dengue occurrence, was determined and characterized.

Historical dengue high risk area also showed a positive association with dengue transmission. This may reaffirm that the area classification based on past history of national surveillance dengue cases has substantial roles in dengue management as well as in providing at-risk areas targeted for the dengue surveillance system and the implementation of dengue control measures. For the variable of having screens for house windows, the presence of house window screens was significantly associated with dengue transmission, compared to the absence of house window screens. Such evidence was consistent with the findings of Chao et al. (2000) [35] while inconsistent with the previous findings of Thammapalo et al. (2012) [36] that households having window screens as a preventive measure showed the reduction of risk in association with human-mosquito contact. In our study, the negative correlation between the presence of house window screens and dengue transmission may result from the cross-sectional survey—where presence of house window screens as an independent variable and houses with a previous history of dengue as dependent variable were measured simultaneously. The existence of many houses that utilize window screens continuously might be due to the perception of dengue and/or due to the health education campaign following dengue outbreaks, whereas the presence of houses with dengue cases was measured after any cases developed within the past year. Accordingly, this finding suggested that the presence of window screens may be dependent upon the particular household's prior dengue risk.

### 3.2. Sociodemographic Risk Factors for Dengue Transmission

Table 3 shows the results of the univariate analysis of sociodemographic risk factors related to dengue transmission considering individual-level characteristics. Using *χ* ^2^ test, 4 out of 22 sociodemographic variables seemed to have a significant association (*P* < 0.05) with frequency of respondents with a history of dengue. Four significant variables were age, highest school degree, residence time in neighborhood, and community effort in environmental management by clean-up campaign, and hence they were selected for further multivariate analysis. Besides these, some variables that either had a significant association at *P* values of nearly 0.05 or had the potentials of significant phenomena were included in the model. The former variables included residence time in the house and number of household members, while the latter variables constituted movement during last 3 months and knowledge about dengue and its vectors. The sociodemographic multivariate analyses revealed findings of the association between 8 individual determinants and dengue transmission (Table 4). 

Among all determinants tested, 5 of them which included age, highest school degree, residence time in neighborhood, number of household members, and community effort in environmental management by clean-up campaign posed significant risk for dengue transmission (the LR test, *P* < 0.05). Respondents aged >45 years showed significant risk for dengue transmission (aOR = 3.24, *P* = 0.003) compared to those aged ≤45 years. With regards to highest school degree, individuals with middle and higher degrees were twice as likely to get dengue infection (aOR = 2.33, *P* = 0.013) compared to those with elementary and lower degrees. In contrast to the univariate analysis, household members >4 persons were likely to experience a greater risk (aOR = 2.01, *P* = 0.02) of dengue transmission than those with ≤4 persons. The last significant predictor was the presence of a community effort in environmental management by clean-up campaign, which was statistically associated with dengue transmission (aOR = 1.91, *P* = 0.035) compared to the absence of community effort.

Sociodemographic factors are commonly targeted for disease prevention and control and underpin successful public health programs [37]. Although there have been promising indications in the literature, some parameters are not well understood in the case of dengue. Risk factors related to dengue transmission are very much influenced by individual and environmental determinants. In this study, individual-level sociodemographic predictors and confounders adjusted for dengue transmission were analyzed through a series of logistic regression models. Older age was associated with higher risk in the area and was in agreement with an earlier study [38]. Furthermore, several other studies have shown that increasing age was significantly associated with dengue transmission [39–41]. Persons who earned secondary and higher degrees of education had a higher risk than those who earned elementary and lower degrees. Regardless of the birth place, this possibly relates to the shorter residence time in neighborhood of respondents that possessed greater risk for dengue transmission compared to the longer residence time. The explanation of such phenomena is that persons who have a high level of schooling may have more chance to get skilled careers. Accordingly, these career opportunities lead to the movement of people seeking jobs far away from their hometown or community, which increases their risk of getting a dengue viral infection from the place where they have moved to work. If infected, these persons could then transmit the dengue virus to their family members and/or others around their homes. Human movement significantly favoring the transmission of dengue correlates well with recent studies [42–44] that have shown this factor to be a major contributor to the acceleration of dengue virus dispersal (and hence disease distribution in space and time), especially between urban/semiurban and rural communities. Human migration allows multiple exposure to *Aedes aegypti *bites among migratory people; in other words, mobile persons have a greater chance of coming into close contact with various bites at multiple locations, especially in public spaces. However, our study did not show a positive association between movements during the previous 3 months and dengue transmission. Larger numbers of household members were more at risk for significant exposure to dengue transmission compared to smaller ones. This finding was supported by the previous study [45] that people gathering with daily activities in a house created the exposure frequency of the bites of dengue-virus infected* Aedes* mosquitoes.

We attempted to evaluate the relative magnitude of knowledge, attitude, and practice of respondents on reducing dengue risk. The knowledge about dengue and its vectors and the attitude about vector control demonstrated no significant association with dengue transmission. For the practice regarding vector control, only the community effort in environmental management by cleaning campaign had a significant effect both in univariate and multivariate analyses. As for the presence of house window screens, this factor was associated with an increase in dengue transmission. Possible explanations are that dengue control efforts by either household members or community participation have generally been performed following the good perception and/or especially during/after dengue outbreaks. In Thailand, dengue prevention and control efforts such as eliminating *Aedes* breeding sites either by individual households or by community participation, personal protection, window screens, and fogging have been intensively applied following the dengue experiences or dengue outbreaks.

## 4. Conclusions

 An integrated analysis of the ecosocial determinants for dengue transmission risk in this study provides meaningful implications and hence it merits the improvement of understanding dynamics of dengue transmission in complex epidemiological settings. First, the ecological analysis model indicated enhanced risk in the RCDENPURA ecotope (i.e., a combination of residential, commercial, and densely populated urban residential areas), in the historical high dengue risk area and in households where screens for windows were present. The ecotope of RCDENPURA and the historical high dengue risk area appear to be very good predictors for dengue incidences. This suggests that dengue control programs could successfully focus on these determinants embedded in the urban ecosystem or elsewhere, especially during an economic crisis and/or when there is a small budget for such programs. Second, the sociological analysis model also revealed plausible significant determinants of older age, higher level of schooling, residence time in the neighborhood, larger household size, and community effort in environmental management by clean-up campaign. Regarding the dynamics of sociodemographic contexts and modern lifestyles, these sociodemographic predictors will significantly help us to understand the processes of dengue transmission dynamics and to implement dengue prevention and control programs effectively and efficiently. Lastly, variances of pertinently ecological and social determinants should be taken into consideration when deliberately formulating local dengue prevention/control programs to gain benefits for both communities and government health practitioners. Meanwhile, these present findings also provide principal grounds for ecosystem and sociodemographic approaches to dengue transmission for researchers in academic institutions. 

## Figures and Tables

**Table 1:** Univariate analysis of the association between ecological factors of households and dengue transmission.

Categorical variables	Household number (%)	Houses (%) with previous history of dengue cases^a^		*P* value
	(*N* = 1,200)	Yes (*n* = 164)	No (*n* = 1,036)
Ecotope^b^				
Commercial	200 (16.7)	23 (14.0)	177 (17.1)	0.031*
DENPURA	200 (16.6)	20 (12.2)	180 (17.4)	
RCDENPURA	200 (16.7)	39 (23.8)	161 (15.5)	
RC	600 (50.0)	82 (50.0)	518 (50.0)	
Historical dengue risk area				
Low	600 (50.0)	57 (34.8)	543 (52.4)	<0.001*
High	600 (50.0)	107 (65.2)	493 (47.6)	
Number of house floors				
One floor	501 (41.8)	70 (42.7)	431 (41.6)	0.861
Multifloors	699 (58.2)	94 (57.3)	605 (58.4)	
Floor of principal living^c^	(*N* = 699)			
First floor	222 (31.8)	34 (36.2)	188 (31.1)	0.385
Upper floors	477 (68.2)	60 (63.8)	417 (68.9)	
Construction material of house				
Concrete/Bricks	1,035 (86.2)	142 (86.6)	893 (86.2)	0.990
Wood	165 (13.8)	22 (13.4)	143 (13.8)	
Number of house windows^d^				
0–9	717 (59.8)	86 (52.4)	631 (60.9)	0.049*
10 and over	483 (40.2)	78 (47.6)	405 (39.1)	
Having screens for house windows^e^	(*N* = 1,139)			
No	407 (35.7)	42 (27.1)	365 (37.1)	0.020*
Yes	732 (64.3)	113 (72.9)	619 (62.9)	
Having a yard/open space				
No	372 (31.0)	41 (25.0)	331 (31.9)	0.090
Yes	828 (69.0)	123 (75.0)	705 (68.1)	
Having bushes in a yard/open space^ f^	(*N* = 828)			
No	454 (54.8)	72 (58.5)	382 (54.2)	0.426
Yes	374 (45.2)	51 (41.5)	323 (45.8)	
Main purpose of house				
Residential	887 (73.9)	126 (76.8)	761 (73.5)	0.413
Business/restaurant	313 (26.1)	38 (23.2)	275 (26.5)	
House attachment				
Attached	977 (81.4)	129 (78.7)	848 (81.9)	0.385
Detached	223 (18.6)	35 (21.3)	188 (18.1)	

^a^Number of houses with at least one dengue case during the past year.

^b^Urban ecotopes were commercial, densely populated urban residential area-DENPURA, and residential mixed with commercial and DENPURA-RCDENPURA, whereas semiurban ecotope was residential mixed with commercial-RC.

Of the 1,200 houses, there were ^c^699 houses that had ≥2 floors; ^d^61 houses that had no windows;^ e^1,139 houses that had at least one window; and ^f^828 houses that had a yard/open space.

*Statistical significance with *χ* ^2^ test (*P* < 0.05) for two-independent samples.

**Table 2:** Multivariate analysis of the association between ecological factors of households and dengue transmission.

Factors	Crude OR (95%CI)	adj. OR (95%CI)	*P* value*	*P* value**
Ecotope				
Commercial	1			0.032**
DENPURA	0.90 (0.48, 1.72)	1.15 (0.58, 2.25)	0.691	
RCDENPURA	1.81 (1.02, 3.21)	2.23 (1.22, 4.07)	0.009*	
RC	1.19 (0.72, 1.97)	1.23 (0.74, 2.05)	0.423	
Historical dengue risk area				
Low	1			
High	2.16 (1.51, 3.09)	2.06 (1.43, 2.95)	<0.001*	<0.001**
Having screens for house windows				
No	1			
Yes	1.59 (1.09, 2.31)	1.62 (1.07, 2.46)	0.023*	0.02**

OR: odds ratios. CI: confidence interval. Statistical significance (*P* < 0.05) using the Wald's test* and the likelihood-ratio test.**

**Table 3:** Univariate analysis of the association between sociodemographic factors of respondents and dengue transmission.

Categorical variables	Respondent number (%)	Respondents (%) with dengue history		*P* value
	(*N* = 1,200)	Yes (*n* = 59)	No (*n* = 1,141)
Age (years)				
≤45	580 (48.3)	10 (16.9)	570 (50)	<0.001*
>45	620 (51.7)	49 (83.1)	571 (50)	
Highest school degree				
Elementary and lower	616 (51.3)	14 (23.7)	602 (52.8)	<0.001*
Secondary and higher	584 (48.7)	45 (76.3)	539 (47.2)	
Occupation				
Unemployed	333 (27.8)	12 (20.3)	321 (28.1)	0.376
Unskilled worker	635 (52.9)	33 (55.9)	602 (52.8)	
Skilled worker	232 (19.3)	14 (23.7)	218 (19.1)	
Movement during last 3 months				
Yes	**79 (6.6)**	7 (11.9)	72 (6.3)	0.159
No	**1,121 (93.4)**	52 (88.1)	1,069 (93.7)	
Residence time in house (years)				
>15	**409 (34.1)**	13 (22.0)	396 (34.7)	0.063
≤15	**791 (65.9)**	46 (78.0)	745 (65.3)	
Residence time in neighborhood (years)				
>15	**524 (43.7)**	14 (23.7)	510 (44.7)	0.002*
≤15	**676 (56.3)**	45 (76.3)	631 (55.3)	
Number of household members (persons)				
1–4	914 (76.2)	39 (66.1)	875 (76.7)	0.088
>4	286 (23.8)	20 (33.9)	266 (23.3)	
Average family income (Baht)				
≤5,000	656 (54.7)	26 (44.1)	630 (55.2)	0.123
>5,000	544 (45.3)	33 (55.9)	511 (44.8)	
Knowledge about dengue and its vectors				
Low	147 (12.2)	11 (18.6)	136 (11.9)	0.183
High	1,053 (87.8)	48 (81.4)	1,005 (88.1)	
Attitude about vector control				
Gender role				
Low/fair	688 (57.3)	33 (55.9)	655 (57.4)	0.930
High	512 (42.7)	26 (44.1)	486 (42.6)	
Family role				
Low	506 (42.2)	28 (47.5)	478 (41.9)	0.478
Fair	694 (57.8)	31 (52.5)	663 (58.1)	
Government role				
Low/fair	506 (42.2)	28 (47.5)	478 (41.9)	0.478
High	694 (57.8)	31 (52.5)	663 (58.1)	
Household water storage				
Yes	1,122 (93.5)	55 (93.2)	1,067 (93.5)	0.856
No	78 (6.5)	4 (6.8)	74 (6.5)	
Household practice to reduce the nuisance of mosquitoes^ a^				
Chemical control				
Low	848 (70.7)	48 (81.4)	800 (70.1)	0.089
High	352 (29.3)	11 (18.6)	341 (29.9)	
Physical control				
Low	667 (55.6)	38 (64.4)	629 (55.1)	0.206
High	533 (44.4)	21 (35.6)	512 (44.9)	
Biological control				
Yes	273 (22.8)	16 (27.1)	257 (22.5)	0.508
No	927 (77.2)	43 (72.9)	884 (77.5)	
Household practices to prevent* Aedes* breeding place^ a^				
Chemical control				
Yes	993 (82.8)	47 (79.7)	946 (82.9)	0.640
No	207 (17.2)	12 (20.3)	195 (17.1)	
Physical control				
Low	270 (22.5)	14 (23.7)	256 (22.4)	0.943
High	930 (77.5)	45 (76.3)	885 (77.6)	
Biological control				
Yes	676 (56.3)	33 (55.9)	643 (56.4)	1.000
No	524 (43.7)	26 (44.1)	498 (43.6)	
Last time visited by health personnel				
Yes (if any time)	856 (71.3)	39 (66.1)	817 (71.6)	0.445
No/don't remember	344 (28.7)	20 (33.9)	324 (28.4)	
Receiving dengue control support/materials				
Yes	934 (77.8)	41 (69.5)	893 (78.3)	0.155
No	266 (22.2)	18 (30.5)	248 (21.7)	
Community effort in environmental				
management by clean-up campaign				
Yes	**513 (42.8)**	16 (27.1)	497 (43.6)	0.019*
No/don't know	**687 (57.2)**	43 (72.9)	644 (56.4)	

^a^Household activities of controlling dengue vector and their assessment were described in the text.

*Statistical significance using *χ* ^2^ test (*P* < 0.05) for two-independent samples.

**Table 4:** Multivariate analysis of the association between sociodemographic factors of respondents and dengue transmission.

Factors	Crude OR (95%CI)	adj. OR (95%CI)	*P* value*	*P* value**
Age (years)				
≤45	1			
>45	4.89 (2.45, 9.75)	3.24 (1.51, 6.97)	0.003*	0.001**
Highest school degree				
Elementary and lower	1			
Secondary and higher	3.59 (1.95, 6.61)	2.33 (1.19, 4.55)	0.013*	0.009**
Movement during last 3 months				
No	1			
Yes	3.59 (0.88, 6.61)	2.33 (0.91, 4.55)	0.080	0.103
Residence time in house (years)				
>15	1			
≤15	1.88 (1.00, 3.52)	0.21 (0.03, 1.35)	0.100	0.054
Residence time in neighborhood (years)				
>15	1			
≤15	2.6 (1.41, 4.79)	6.19 (1.03, 37.26)	0.047*	0.011**
Number of household members (persons)				
1–4	1			
>4	1.69 (0.97, 2.94)	2.01 (1.12, 3.6)	0.020*	0.024**
Knowledge about dengue and its vectors				
High	1			
Low	1.69 (0.86, 3.34)	1.9 (0.93, 3.88)	0.080	0.096
Community effort in environmental				
management by clean-up campaign				
No/don't know	1			
Yes	2.07 (1.15, 3.73)	1.91 (1.05, 3.49)	0.035*	0.029**

OR: odds ratios. CI: confidence interval. Statistical significance (*P* < 0.05) using the Wald's test* and the likelihood-ratio test.**
